# Significance of Triple Detection of p16/ki-67 Dual-Staining, Liquid-Based Cytology and HR HPV Testing in Screening of Cervical Cancer: A Retrospective Study

**DOI:** 10.3389/fonc.2022.915418

**Published:** 2022-06-07

**Authors:** Li Yu, Xun Chen, Xubin Liu, Lingyan Fei, Hanyu Ma, Tian Tian, Liantang Wang, Shangwu Chen

**Affiliations:** ^1^ Department of Pathology, The First Affiliated Hospital, Sun Yat-sen University, Guangzhou, China; ^2^ Hospital of Stomatology, Guanghua School of Stomatology, Guangdong Provincial Key Laboratory of Stomatology, Sun Yat-sen University, Guangzhou, China; ^3^ State Key Laboratory for Biocontrol, Guangdong Key Laboratory of Pharmaceutical Functional Genes, Key Laboratory of Gene Engineering of the Ministry of Education, Department of Biochemistry, School of Life Sciences, Sun Yat-sen University, Guangzhou, China

**Keywords:** triple detection, screening of cervical cancer, p16/ki-67 dual-staining, liquid-based cytology, HR HPV testing

## Abstract

In addition to liquid-based cytology (LBC) and HR HPV testing, p16/ki-67 dual-staining is another method for cervical cancer screening. The combination of any two methods can improve the accuracy of screening, but some cervical lesions are still missed or misdiagnosed. In this retrospective study, the significance of LBC, HR HPV testing and especially p16/ki-67 dual-staining in cervical lesion screening was evaluated with reference to histological diagnosis. At the same time, we tried to explore the value of p16/ki-67 dual-staining combined with LBC and HR HPV testing (triple detection) in improving the diagnostic specificity of CIN2+ and reducing the missed diagnosis of CIN2+ lesions. We found that p16/ki-67 dual-staining was valuable in identifying cervical CIN2+ lesions and reducing the missed diagnosis of CIN2+ in HPV negative patients. More than 96% of CIN2+ patients were positive for two or three tests of triple detection. Whole positive triple detection can effectively predict high grade cervical lesions. In conclusion, the triple detection can distinguish almost all cervical CIN2+ lesions. Our data put forward and highlight the feasibility and significance of triple detection in cervical lesion screening.

## Introduction

Cervical cancer is one of the most common female malignant tumors worldwide, and human papillomavirus (HPV) infection is essential cause. Cervical cancer screening is valuable to early find out uterine precancerous lesions. Timely treatment of these lesions may prevent or avoid the occurrence of cervical cancer. At present, cervical cancer screening mainly adopts three strategies, including liquid-based cytology (LBC), HPV testing with partial genotyping, as well as the combined application of the above two methods.

Each method has its own advantages and disadvantages. Cytology screening is one of the earliest and most widely used methods, which is characterized by high specificity and relatively low sensitivity. When diagnosed by LBC, about 4-8% atypical squamous cells of undetermined significance (ASCUS) and 12-15% low-grade squamous intraepithelial lesions (LSIL) are grade 2 or more severe cervical intraepithelial neoplasia (CIN2+) lesions, which need to be triaged (1). How to distinguish LSIL from high-grade squamous intraepithelial lesions (HSIL), discriminate atrophy, metaplasia and HSIL, and improve detection rate of glandular epithelial lesions are still urgent problems to be solved in cytology screening (2). HPV testing has high sensitivity and high negative predictive value (NPV), which can overcome some shortcomings of cytology screening methods, but its specificity is low compared with LBC (3–5). In addition, HPV testing cannot distinguish HPV transient infection from precancerous lesions. HPV infection is usually temporary, and the virus will be eliminated in a few months to years. Only a low proportion of infections persists and may develop into HSIL (6). Therefore, women with high-risk HPV (HR HPV) infection should be further triaged even if their cytological appearance is normal. American FDA guidelines require that HPV16/18 positive patients undergo colposcopy immediately, while HPV positive but HPV16/18 negative women undergo cytology. If cytology is negative, follow up will be performed after 12 months (7).

In order to overcome the shortcomings of HPV and cytology screening, find precancerous lesions as early as possible and reduce the referral rate of colposcopy, it is necessary to find other biomarkers with high sensitivity and specificity. p16/ki-67 dual-staining cytology was reported to be an alternative method in cervical cancer screening (8–13). p16 (p16INK4A) is encoded by CDKN2A and is an important regulator of cell cycle (14). As a tumor suppressor protein, down-regulation of p16 expression is usually associated with increased cancer risk (15). However, indirect activation of cell cycle by HPV E7 oncoprotein induces overexpression and accumulation of p16 through a negative feedback loop (16). Therefore, the expression of p16 in cervical tissues is closely related to HR HPV infection and is regarded as a surrogate marker for persistent HR HPV infection (17). ki-67 is a cell proliferation marker, which can predict the malignant potential of tumors and is an important index for prognosis and prediction of many tumors (18). The detection of ki-67 expression has been widely used in the auxiliary diagnosis of cervical precancerous lesions and cancer (19). In physiological situations of the cervical epithelial cells, the over-expression of p16 and the expression of ki-67 are mutually exclusive. The p16/ki-67 co-expression implies deregulation of the cell cycle induced by HR HPV. Detection of p16/ki-67 co-expression can be used as a marker to predict HR HPV mediated cell transformation and high grade CIN lesions. The sensitivity of p16/ki-67 dual-staining was usually lower than that of HPV testing, and the specificity was comparable to that of LBC (9). However, when p16/ki-67 dual-staining was used to triage HPV positive (20–24), HR HPV+/NILM (22, 25, 26), ASCUS and LSIL (22, 27–30), it showed relative high sensitivity and specificity (2). In our previous study, we found that p16 and ki-67 immunostaining on cell block preparations can improve the diagnostic accuracy of HSIL and squamous cell carcinoma (31).

Although different screening methods or method combinations have been used to early identify cervical epithelial lesions or cervical cancer, some patients are still missed or misdiagnosed. In this retrospective study, we evaluated the significance of p16/ki-67 dual-staining in cervical cancer screening and the value of triple detection (p16/ki-67 dual-staining combined with LBC and HPV testing) in improving the specificity of CIN2+ diagnosis and reducing the missed diagnosis of CIN2+ lesions by comparing the coincidence of three examinations, including LBC, HPV testing and p16/ki-67 dual-staining, with histological diagnosis.

## Materials and Methods

### Participants

**Figure 1:**
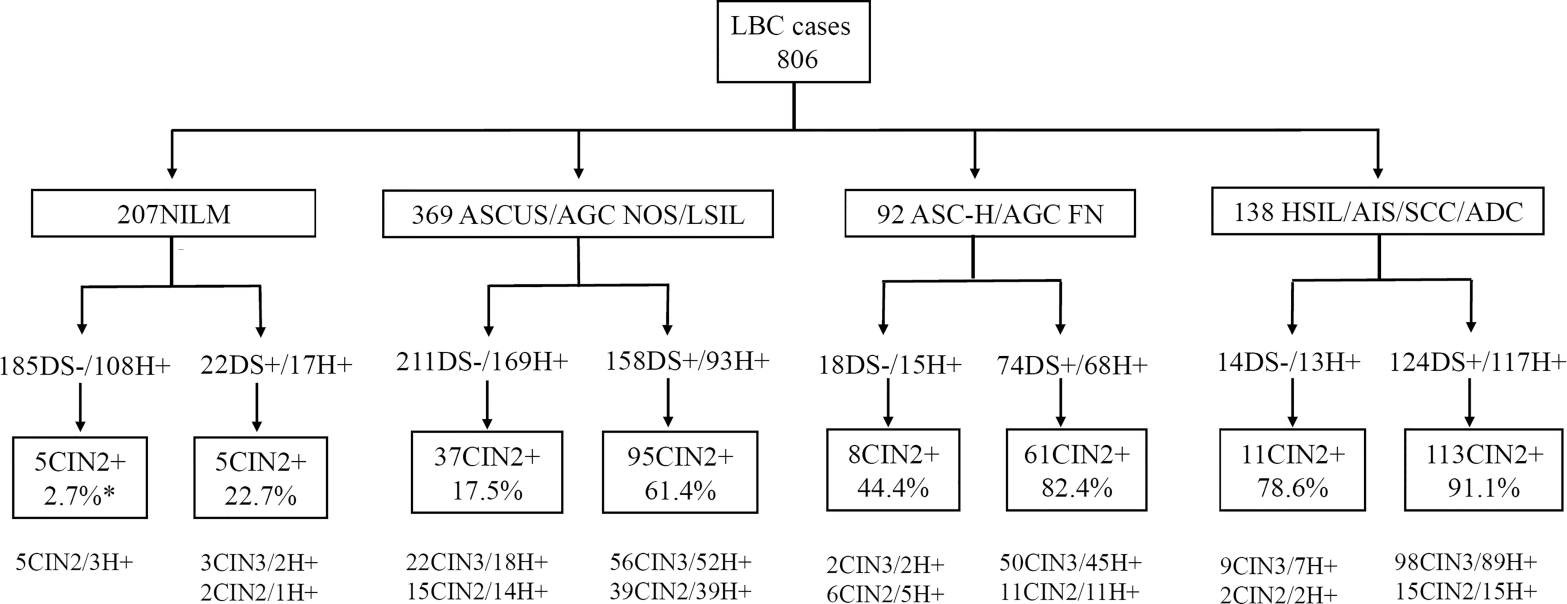
Graphic overview of patient test results in this study. *CIN2+ cases/DS positive or negative cases. DS, p16/ki-67 dual-staining; H+, positive for HR HPV; LBC, liquid-based cytology; ADC, adenocarcinoma; AGC FN, atypical glandular cells, favor neoplastic; AGC NOS, atypical endocervical cells, not otherwise specified; AIS, adenocarcinoma *in situ*; ASC-H, atypical squamous cells cannot exclude high-grade squamous intraepithelial lesion; ASCUS, atypical squamous cells of undetermined significance; CIN, cervical intraepithelial neoplasia; CIN2+, cervical intraepithelial neoplasia grade 2 or worse; CIN3+, cervical intraepithelial neoplasia grade 3 or worse; HSIL, high-grade squamous intraepithelial lesion; LSIL, low-grade squamous intraepithelial lesion; NILM, negative for intraepithelial lesions or malignancy; SCC, squamous cell carcinoma.

This retrospective study involved 806 patients selected form the Department of Pathology, the First Affiliated Hospital, Sun Yet-sen University from January 2015 to December 2020 (**Figure 1**). All patients completed Pap cytology, HR HPV testing, p16/ki-67 dual-staining cytology, and histopathological diagnosis. Tissue specimens were obtained by colposcopy or hysterectomy. Most patients were referred to colposcopy due to cytological abnormalities or HPV positive, while a few of women underwent a total hysterectomy for endometriosis or uterine leiomyoma. The study was approved by the hospital ethics committee, and all patients and controls were informed and consented before their participation in the study.

### Liquid-Based Cytology (LBC)

Thin-layer cytology slides were prepared with SurePath Pap Test (BD Diagnostics, Burlington, NC) and stained by the Papanicolaou method. Cervical cytology was independently interpreted by cytopathologists and classified according to the Bethesda 2015 classification system (2). Two experienced cytopathologists reviewed all cytological slides and approved the final report. The LBC results were defined as negative for intraepithelial lesions or malignancy (NILM), atypical squamous cells of undetermined significance (ASCUS), atypical endocervical cells, not otherwise specified (AGC NOS), low-grade squamous intraepithelial lesion (LSIL), atypical squamous cells cannot exclude high-grade squamous intraepithelial lesion (ASC-H), atypical glandular cells, favor neoplastic (AGC FN), high-grade squamous intraepithelial lesion (HSIL), adenocarcinoma *in situ* (AIS), squamous cell carcinoma (SCC), and adenocarcinoma (ADC). Except for NILM, other cervical lesions were defined as ASCUS+ or positive. After liquid-based cytology, the residual cytological materials were used for HR HPV testing and p16/ki-67 immunostaining.

### HPV Testing

In this study, HR HPV was detected in 371 patients by Cobas HPV Test (Roche Molecular Systems Inc. Pleasanton, CA) and 435 patients by Hybrid Capture 2 (HC2) HPV Test (Qiagen, Gaithersburg, MD), respectively. Cobas HPV Test is able to detect HPV16 and HPV18 individually and other 12 pooled HR HPV genotypes (31, 33, 35, 39, 45, 51, 52, 56, 58, 59, 66 and 68). HC2 system can detect 13 pooled HR HPV genotypes (16, 18, 31, 33, 35, 39, 45, 51, 52, 56, 58, 59 and 68), and a relative light unit of 1 (1.0 pg/mL) was used as the cut off for HR HPV positivity. All procedures were carried out in accordance with the manufacturer’s instructions.

### p16/ki-67 Dual-Staining Cytology

**Figure 2:**
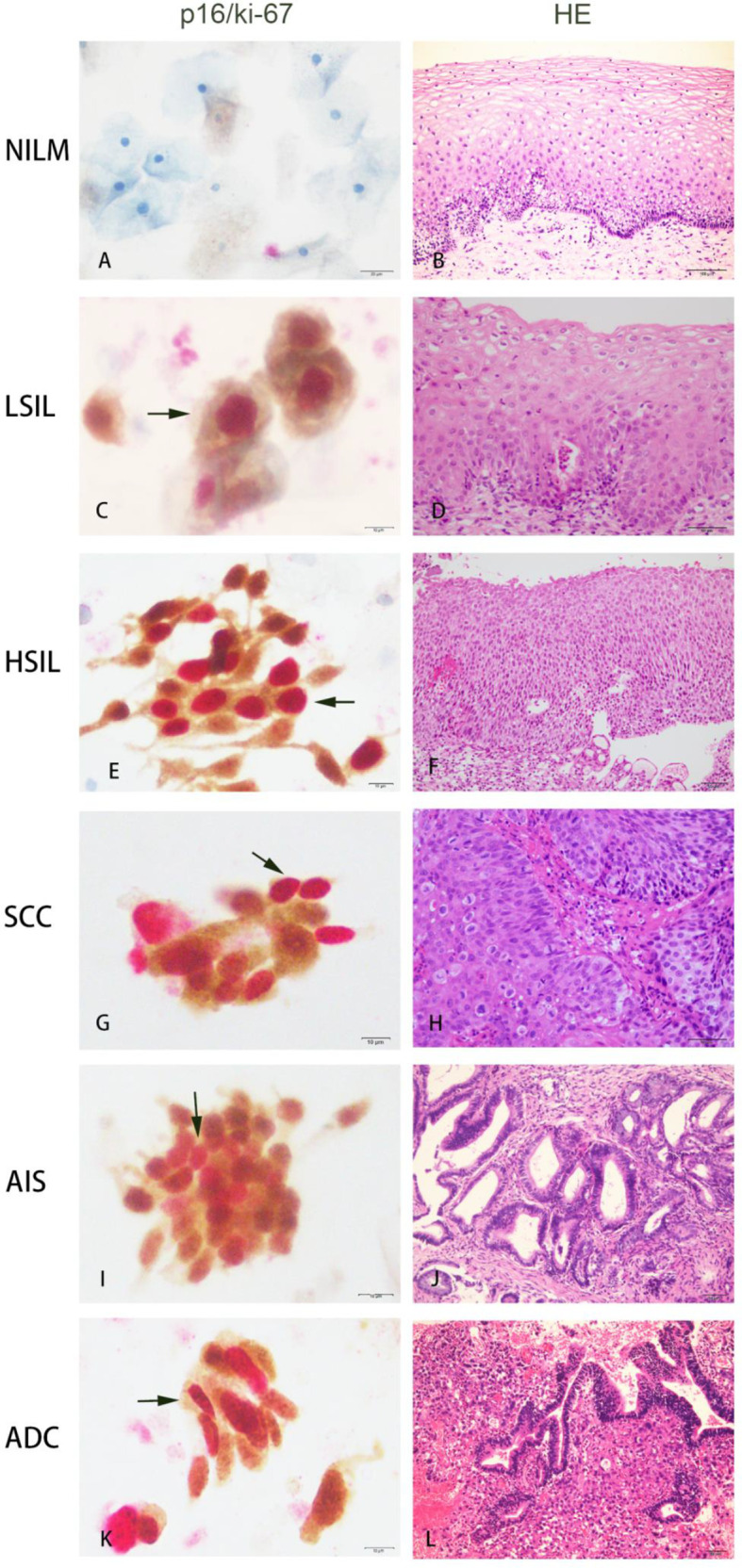
Co-expression of p16/ki-67 in cytological specimens detected by p16/ki-67 dual-staining (DS) and haematoxylin and esion (HE) staining in histology of the same cases. The positive p16/ki-67 dual-staining cells (dark arrow) are characterized by a brown cytoplasmic signal for p16 overexpression and a dark red nuclear signal for p16/ki-67 co-expression in the same cells. NILM, negative for intraepithelial lesions or malignancy; LSIL, low-grade squamous intraepithelial lesion; HSIL, high-grade squamous intraepithelial lesion; SCC, squamous cell carcinoma; AIS, adenocarcinoma *in situ*; ADC, adenocarcinoma.

Immunostaining for p16 and ki-67 expression was performed on cytological specimens using the CINtec Plus kit and VENTANA BenchMark XT automated slide stainers (Roche Tissue Diagnostics/Ventana Medical Systems, Inc., Tucson, AZ) according to manufacturer’s instructions. The primary antibody cocktail comprises a mouse monoclonal antibody (clone E6H4) against human p16 protein and a rabbit monoclonal antibody (clone 274-11 AC3) against human ki-67 protein. Horseradish peroxidase-mediated conversion of 3,3-diaminobenzidine (DAB) and alkaline phosphatase-mediated conversion of Fast Red resulted in brown cytoplasmic/nuclear staining at p16 antigen sites and red nuclear staining at ki-67 antigen sites, respectively. p16/ki-67 dual-staining cells showed brown cytoplasm signals for p16 expression and dark red to red brown nuclear signals for the co-expression of p16 and ki-67 in the same cell. The presence of one or more p16/ki-67 dual-staining cervical epithelial cells was defined as a positive result, regardless of cell morphology (**Figure 2**). Samples without any dual-staining cells were determined to be negative for p16⁄ki-67 dual-staining.

### Histopathology

Tissue samples were collected by colposcopy and hysterectomy and processed according to standard histological procedures. The sections were independently diagnosed by the pathologists according to the classification of the 2014 WHO guidelines for cervical histopathology (32). Final histopathological reports were reviewed and approved by two senior pathologists. In this study, high-grade squamous intraepithelial lesion (HSIL) (including CIN2 and CIN3), squamous cell carcinoma (SCC), adenocarcinoma *in situ* (AIS) and adenocarcinoma (ADC) were referred to as CIN2+; CIN3, SCC, AIS and ADC were referred to as CIN3+; and Negative (absence of lesions or presence of benign alteration including reactive alterations, squamous metaplasia and atrophy) and CIN 1 were referred to as CIN2-.

### Statistical Analysis

Chi square of trend for proportion was calculated to test linear associations between screening methods and increasing severity of cytological and histological diagnoses. Associations between p16/ki-67 expression and HR HPV positivity were examined using logistic regression models. Sensitivity [true positive/(true positive + false negative)], specificity [true negative/(true negative + false positive)], positive predictive value [PPV, true positive/(true positive + false positive)] and negative predictive value [NPV, true negative/(true negative + false negative)] were calculated for 2 different endpoints, CIN2+ and CIN3+. Estimates were provided with their 95% confidence intervals (95% CI). In addition, area under ROC curve (AUC) and referral rates to colposcopy based on test positivity were calculated. McNemar tests were used to compare paired matching data such as sensitivities, specificities, PPV and NPV between different screening methods. Analyses were performed in R, version 3.3.1. All *P* values were from 2-sided tests and results were deemed statistically significant at *P* < 0.05.

## Results

A total of 806 women were enrolled in our study. They all completed LBC, HR HPV testing and p16/ki-67 dual-staining, which were confirmed by histological diagnosis. Their average age was 42.4 ± 10.6 years, ranging from 19 to 81 years, with a median of 41 years. They were respectively diagnosed as the Negative, CIN1, CIN2, CIN3, AIS, SCC and ADC in histology or categorized as NILM, ASCUS, AGC NOS, LSIL, ASC-H, AGC FN, HSIL, AIS, SCC and ADC in cytology (**Figure 1**; **Table 1**).

**Table 1:** Positivity of p16/ki-67, cytology and HR HPV in histology and cytology categories.

Categories	Cases	p16/ki-67, n (%)	HR HPV, n (%)	Cytology/ASCUS+, n (%),
**Histology**	**806**	**378 (46.8)**	**659 (81.8)**	**599 (74.3)**
Negative	216	33 (15.3)	142 (65.8)	80 (30.0)
CIN1	255	61 (24.0)	205 (80.4)	194 (76.1)
CIN2	95	73 (76.8)	91 (95.8)	88 (92.6)
CIN3	164	142 (86.6)	152 (92.7)	161 (98.2)
AIS	7	5 (71.4)	6 (85.7)	7 (100.0)
SCC	48	45 (93.8)	45 (93.8)	48 (100.0)
ADC	21	19 (90.5)	18 (85.7)	21 (100.0)
*P* for trend		< 0.001	< 0.001	< 0.001
CIN2+	335	284 (84.8)	312 (93.1)	325 (97.0)
CIN3+	240	211 (87.9)	221 (92.1)	237 (98.8)
**Cytology**	**806**	**378 (46.8)**	**659 (81.8)**	
NILM	207	22 (10.8)	125 (60.4)	
ASCUS	167	76 (45.5)	146 (87.4)	
AGC NOS	10	2 (20.0)	5 (50.0)	
LSIL	192	80 (41.7)	173 (90.1)	
ASC-H	78	60 (76.9)	68 (87.2)	
AGC FN	14	14 (100.0)	13 (92.9)	
HSIL	123	110 (89.4)	116 (94.3)	
AIS	1	1 (100.0)	1 (100.0)	
SCC	11	11 (100.0)	10 (90.9)	
ADC	3	2 (66.7)	2 (66.7)	
*P* for trend		< 0.001	< 0.001	

ADC, adenocarcinoma; AGC FN, atypical glandular cells, favor neoplastic; AGC NOS, atypical endocervical cells, not otherwise specified; AIS, adenocarcinoma in situ; ASC-H, atypical squamous cells cannot exclude high-grade squamous intraepithelial lesion; ASCUS, atypical squamous cells of undetermined significance; ASCUS+, other cervical cytological lesions except for NILM; CIN, cervical intraepithelial neoplasia; CIN2+, cervical intraepithelial neoplasia grade 2 or worse; CIN3+, cervical intraepithelial neoplasia grade 3 or worse; HSIL, high-grade squamous intraepithelial lesion; LSIL, low-grade squamous intraepithelial lesion; NILM, negative for intraepithelial lesions or malignancy; SCC, squamous cell carcinoma. Bold values indicate totals.

### Positive Rates of p16/ki-67 Dual-Staining in Cytology and Histology Categories

We first analyzed the positive rates of p16/ki-67 dual-staining and HR HPV in histology or cytology categories and the positive rates of LBC in histology category (**Table 1**). p16/ki-67 positive rate significantly increased with the severity of the cytological lesions from 10.8% (22/207) in NILM to 92.9% (13/14) in patients with cancers (SCC+ADC) (*P*trend < 0.001), and with severity of the histological lesions from 15.3% (33/216) in Negative to 91.3% (63/69) in patients with cancers (SCC+ADC) (*P*trend < 0.001).

### Association of p16/ki-67 Dual-Staining With HR HPV Infection

The association of HR HPV infection with p16/ki-67 dual-staining was evaluated. According to HPV DNA testing results, 806 patients were divided into HR HPV negative group and positive group. The positive rate of p16/ki-67 dual-staining in HR HPV positive samples significantly higher than that in HR HPV negative samples (*p* < 0.001) (**Table 2**). When the cases were stratified as CIN2- and CIN2+ by histology, the association was still significant (*p*=0.002 and *p*=0.042). To analyze the correlation between HPV genotypes and p16/ki-67 dual-staining, 371 cases with partial HPV genotyping were grouped into HPV16/18 positive, other 12 HR HPV types positive, and HR HPV negative. The positive rates of p16/ki-67 dual-staining in HPV16/18 positive (OR 14.6, 95% CI: 6.9-31.0) and other 12 HR HPV types positive (OR 5.0, 95% CI: 2.5-9.8) were significantly higher than those in HR HPV negative (*p* < 0.001). When the cases were stratified by histology, the positive rate of p16/ki-67 dual-staining in CIN2- group was significantly different (*p*=0.006 and *p*=0.020), but there was no significant difference in CIN2+ group (*p*=0.300 and *p*=0.254) (**Table 2**).

**Table 2:** Association of p16/ki-67 dual-staining with HR HPV infection and HR HPV genotypes in histology category.

Cases	HR HPV	p16/ki-67+ n (%)	p16/ki-67- n (%)	*P* Value	OR (95% CI)
**Total n=806**					
	HR HPV negative (*n* = 147)	41 (27.9)	106 (72.1)		
	HR HPV positive (*n* = 659)	315 (47.8)	344 (52.2)	<0.001	4.8 (3.1-7.5)
CIN2- n=471					
	HR HPV negative (*n* =124)	11 (8.9)^a^	113 (91.1)		
	HR HPV positive (*n* = 347)	75 (21.6)	272 (78.4)	0.002	2.8 (1.5-5.5)
CIN2+ n=335					
	HR HPV negative (*n* = 23)	16 (69.6)^b^	7 (30.4)		
	HR HPV positive (*n* = 312)	268 (85.9)	44 (14.1)	0.042	2.7 (1.0-6.9)
**Cobas n=371**					
	HR HPV negative (*n* = 75)	12 (16.0)	63 (84.0)		
	HPV16/18 positive (*n* = 107)	78 (72.9)	30 (28.0)	<0.001	14.6 (6.9-31.0)
	Other 12 positive (*n* = 189)	91 (48.2)	98 (51.9)	<0.001	5.0 (2.5-9.8)
CIN2- n=209					
	HR HPV negative (*n* = 65)	5 (7.7)	60 (92.3)		
	HPV16/18 positive (*n* = 29)	9 (31.0)	20 (69.0)	0.006	5.4 (1.6-18.0)
	Other 12 positive (*n* = 115)	25 (21.8)	90 (78.3)	0.020	3.3 (1.2-9.2)
CIN2+ n=162					
	HR HPV negative (*n* = 10)	8 (80.0)	2 (20.0)		
	HPV16/18 positive (*n* = 78)	70 (89.7)	8 (10.3)	0.300	2.5 (0.7-16.9)
	Other 12 positive (n =74)	68 (91.9)	6 (8.1)	0.254	2.8 (0.5-16.0)

CI, confidence interval; CIN2+, cervical intraepithelial neoplasia grade 2 or worse; OR, odds ratio.

Other 12 positive: positive for 12 HPV genotypes (HPV 31, 33, 35, 39, 45, 51, 52, 56, 58, 59, 66 and 68), and negative for HPV16/18.

a VS b, P<0.001. Bold values indicate totals.

### Sensitivity, Specificity, PPV, NPV, and AUC of Individual Method or Combined Application

We calculated the sensitivity, specificity, positive predictive value (PPV), negative predictive value (NPV), and area under ROC curve (AUC) of p16/ki-67, HR HPV and cytology as well as three different combinations of these methods in screening for CIN2+ or CIN3+ (**Table 3**). The sensitivity of cytology, HR HPV and p16/ki-67 for CIN2+ were 97.0%, 93.1%, and 84.8%, respectively. The sensitivity of triple detection was 79.1%, which was the lowest among various screening methods, comparable to the sensitivity (80.0%, *p*=0.780, **Table S1**) of p16/ki-67 combined with HR HPV. However, the specificity and PPV of triple detection were 86.8% and 81.0%, respectively, which was the highest in the single or combined applications of the three methods except for p16/ki-67 with HR HPV (*p*=0.254 and *p*=0.366) and PPV of p16/ki-67 (*p*=0.186). The specificity and PPV of p16/ki-67 alone was 81.7% and 76.8%, respectively, which was higher than 41.8% and 54.3% in cytology (*p* < 0.001) and 26.3% and 47.3% in HR HPV (*p* < 0.001). The NPV of cytology was 95.2%, which was the highest among various methods (all *p* < 0.05). Among all analytical methods, AUC of p16/ki-67 alone or triple detection was the highest. The change trend of these parameters also existed in CIN3+ patients.

**Table 3:** Performance of p16/ki-67 dual-staining, cytology and HR HPV testing in detection of CIN2+ or CIN3+.

Methods	Sensitivity	Specificity	PPV	NPV	AUC
**CIN2+ n=335**					
p16/ki-67 n=284	84.8 (80.4-88.4)*	81.7 (77.9-85.1)	76.8 (72.1-80.9)	88.3 (84.8-91.1)	83.3 (80.7-85.9)
Cytology n=325	97.0 (94.4-98.5)	41.8 (37.3-46.4)	54.3 (50.2-58.3)	95.2 (91.0-97.5)	69.4 (67.0-71.8)
HR HPV n=312	93.1 (89.7-95.5)	26.3 (22.5-30.6)	47.3 (43.5-51.2)	84.4 (77.2-89.6)	59.7 (57.3-62.1)
^a^DSH+ n=268	80.0 (75.2-84.1)	84.1 (80.4-87.2)	78.1 (73.3-82.3)	85.5 (81.9-88.5)	82.0 (79.3-84.8)
^b^CH+ n=306	91.3 (87.7-94.0)	52.2 (47.6-56.8)	57.6 (53.3-61.9)	89.5 (85.1-92.0)	71.8 (69.1-74.5)
^c^DSHC+ n=265	79.1 (74.3-83.3)	86.8 (83.4-89.7)	81.0 (76.3-85.1)	85.4 (81.8-88.4)	83.0 (80.3-85.6)
**CIN3+ n=240**					
p16/ki-67 n=211	87.9 (83.0-91.6)	71.9 (68.0-75.5)	57.0 (51.8-62.1)	93.4 (90.5-95.4)	79.9 (77.1-82.7)
Cytology n= 237	98.8 (96.1-99.7)	36.0 (32.1-40.2)	39.6 (35.7-43.6)	98.6 (95.5-99.6)	67.4 (65.3-69.5)
HR HPV n=222	92.5 (88.2-95.4)	22.8 (19.4-26.5)	33.7 (30.1-37.5)	87.8 (81.1-92.4)	57.7 (55.2-60.1)
DSH+ n=197	82.1 (76.5-86.6)	74.2 (70.4-77.7)	57.4 (52.0-62.7)	90.7 (87.6-93.1)	78.1 (75.1-81.2)
CH+ n=220	91.7 (87.2-94.7)	45.1 (40.9-49.3)	41.4 (37.2-45.8)	92.7 (88.8-95.4)	68.4 (65.7-71.1)
DSHC+ n=195	81.2 (75.6-85.9)	76.7 (73.0-80.0)	59.6 (54.1-64.8)	90.6 (87.5-93.0)	79.0 (75.9-82.0)

a DSH, p16/ki-67, HR HPV;

b CH, cytology, HR HPV;

c DSHC, p16/ki-67, HR HPV, cytology.

*% (95% CI).

AUC, area under ROC curve; CI, confidence interval; CIN2+, cervical intraepithelial neoplasia grade 2 or worse; CIN3+, cervical intraepithelial neoplasia grade 3 or worse; NPV, negative predictive value; PPV, positive predictive value. Bold values indicate totals.

### p16/ki-67 Dual-Staining Is Valuable for the Identification of CIN2+, Regardless of HPV Genotype and Tissue Type

We further analyzed the relationship between HPV genotypes and severity of cervical squamous or glandular epithelial lesions as well as the positive rate of p16/ki-67 (**Table 4**). HPV16 was the most dominant HPV genotype in cervical squamous carcinoma (56.3%), while other 12 HR HPV genotypes rather than HPV16/18, were predominant in various CINs. The positive rate of HPV16 increased and the positive rate of other 12 HR HPV genotypes decreased with the severity of lesions from CIN2, CIN3 to SCC, but the positive rate of p16/ki-67 in all HPV positive cases was relatively high and comparable (**Table 4**). The most dominant HPV genotype in AIS and ADC was HPV18 (42.9% and 33.3%) and HPV16 (28.6% and 33.3%). The p16/ki-67 positive rate of all HPV16/18 positive glandular epithelial lesions was 89.5% (17/19), which was comparable to that of squamous CIN2+ lesions. Similarly, the positive rate of p16/ki-67 was 87.5% (14/16) in the CIN2+ cases co-infected with any two of HPV16, HPV18 and other 12 HR HPV genotypes. The data suggested that the positive rate of p16/ki-67 was related to the severity of cervical lesions and whether they were infected with HPV, regardless of HPV genotype (*P*>0.05, **Table 2**) and tissue type.

**Table 4:** Relationship of HPV genotypes and severity of cervical lesions and the positive rate of p16/ki-67.

HPV genotype/DS	Cases (n=371)	Negative (n=110)	CIN1 (n=99)	CIN2 (n=41)	CIN3 (n=61)	SCC (n=32)	AIS (n=7)	ADC (n=21)
HPV16+	70 (18.9)	11 (10.0)	8 (8.1)	6 (16.7)	18 (29.5)	18 (56.3)	2 (28.6)	7 (33.3)
HPV16+, DS+	53/70 (75.7)	4/11 (36.4)	5/8 (62.5)	5/6 (83.3)	15/18 (83.3)	17/18 (94.4)	1/2 (50.0)	7/7 (100.0)
HPV18+	21 (5.7)	6 (5.5)	3 (3.0)	1 (2.4)	0	1 (3.1)	3 (42.9)	7 (33.3)
HPV18+, DS+	11/21 (52.4)	0	0	1/1 (100.0)	0	1/1 (100.0)	3/3 (100.0)	6/7 (85.7)
HPV16+/18+	3 (0.8)	0	0	0	2 (3.3)	0	0	1 (4.8)
HPV16+/18+, DS+	3/3 (100.0)	0	0	0	2/2 (100.0)	0	0	1/1 (100.0)
HPV16+/other 12+	11 (3.0)	0	1 (1.0)	3 (7.3)	6 (9.8)	1 (3.1)	0	0
HPV16/other12+, DS+	9/11 (81.8)	0	0	2/3 (66.7)	6/6 (100.0)	1/1 (100.0)	0	0
HPV18+/other 12+	2 (0.5)	0	0	0	0	0	0	2 (9.5)
HPV18+/other 12+, DS+	2/2 (100.0)	0	0	0	0	0	0	2/2 (100.0)
Other 12+	189 (50.9)	55 (50.0)	60 (60.6)	30 (73.2)	32 (52.5)	10 (31.3)	1 (14.3)	1 (4.8)
Other 12+, DS+	91/189 (48.2)	13/55 (23.6)	12/60 (20.0)	29/30 (96.7)	28/32 (87.5)	9/10 (90.0)	1/1 (100.0)	1/1 (100.0)
HR HPV-	75 (20.2)	38 (34.6)	27 (27.3)	1 (2.4)	3 (4.9)	2 (6.3)	1 (14.3)	3 (14.3)
HPV-, DS+	12/75 (16.0)	3/38 (7.9)	3/27 (11.1)	1/1 (100.0)	3/3 (100.0)	2/2 (100.0)	0	2/3 (66. 7)

CIN, cervical intraepithelial neoplasia; DS, p16/ki-67 dual-staining; HPV, human papillomavirus.

Other 12+: positive for 12 HR HPV genotypes (31, 33, 35, 39, 45, 51, 52, 56, 58, 59, 66 and 68) except for HPV16/18.

### p16/ki-67 Dual-Staining Is Helpful to Reduce the Missed Diagnosis of CIN2+ in HPV Negative Patients

Of a total 806 patients, 147 were HPV negative, including 124 CIN2- and 23 CIN2+ (**Table 2**). A few HPV negative CIN2- cases were detected p16/ki-67 positive (8.9%, 11/124), while about 70% (16/23) of HPV negative CIN2+ was positive for p16/ki-67 dual-staining (**Table 2**). There was significant difference in the positive rate of p16/ki-67 between two groups (*p* < 0.001). Obviously, the positive rate in HPV negative CIN2+ cases is higher than that in HPV negative CIN2- cases.

### Triple Detection Showed the Great Advantages in Screening of CIN2+ Lesions

When the triple detection was used to screen CIN2+ and CIN3+, the cases positive for all three methods was 80.0% (268/355) and 82.1% (197/240), respectively (**Table 5**). In the remaining patients, 16.4% of CIN2+ and 15.8% of CIN3+ were positive in at least any two methods, and 3.0% of CIN2+ and 2.1% of CIN3+ were positive in any one detection. That is, more than 96% of CIN2+ patients were positive in two or more detections. In triple detection, only two CIN2+ patients were negative and none of CIN3+ patients were negative.

**Table 5:** Triple detection with p16/ki-67 dual-staining, cytology, and HPV testing.

Histology	Cases	Cytology (C), p16/ki-67 dual-staining (D), HPV (H) testing. n (%)							
		C+ D+ H+	H- D- C-	D- (C+ H+)	D+ (H- C-)	H- (C+ D+)	H+ (D- C-)	C- (D+ H+)	C+ (D- H-)
SCC, ADC	69	59 (87.0)	0	4 (4.3)	0	5 (7.3)	0	0	1 (1.5)
HSIL (CIN3/AIS)	171	138 (80.7)	0	19 (11.1)	1 (0.6)	9 (5.3)	0	1 (0.6)	3 (1.8)
HSIL (CIN2)	95	71 (74.7)	2 (2.1)	16 (16.8)	1 (1.1)	0	3 (3.2)	1 (1.1)	1 (1.1)
LSIL	255	45 (17.7)	18 (7.1)	120 (47.1)	2 (0.8)	4 (1.6)	36 (14.1)	4 (1.6)	26 (10.2)
Negative	216	19 (8.8)	53 (24.5)	42 (19.4)	2 (0.9)	3 (1.4)	73 (33.8)	8 (3.7)	16 (7.4)
Total	806	332 (41.2)	73 (9.1)	201 (24.9)	6 (0.7)	21 (2.6)	112 (13.9)	14 (1.7)	47 (5.8)
CIN2+	335	268 (80.0)	2 (0.6)	39 (11.6)	2 (0.6)	14 (4.2)	3 (0.9)	2 (0.6)	5 (1.5)
CIN3+	240	197 (82.1)	0	23 (9.6)	1 (0.4)	14 (5.8)	0	1 (0.4)	4 (1.7)

ADC, adenocarcinoma; AIS, adenocarcinoma in situ; CIN, cervical intraepithelial neoplasia; CIN2+, cervical intraepithelial neoplasia grade 2 or worse; CIN3+, cervical intraepithelial neoplasia grade 3 or worse; HSIL, high-grade squamous intraepithelial lesion; LSIL, low-grade squamous intraepithelial lesion; SCC, squamous cell carcinoma.

### Positive Triple Detection May Predict the Potential High Grade Cervical Lesions

When reviewing the patients’ medical history, we found that 8 patients were positive for HR HPV and p16/ki-67 dual-staining (**Table 6**). They were diagnosed as ASCUS+ in cytology, but all lacked HSIL characteristics histologically. Seven patients underwent another biopsy and one patient underwent three additional biopsies. Six of them were finally confirmed as CIN2+, and two patients were diagnosed as vaginal intraepithelial neoplasia (VaIN) II and VaIN III, respectively. Another patient was initially diagnosed as HSIL by cytology. Her p16/ki-67 dual-staining was positive, but HR HPV was negative. She was followed up for 52 months and confirmed as CIN3+.

**Table 6:** Patients positive for p16/ki-67 and cytology, but no CIN 2+ characteristics in histology.

Cases	Age	Cytology	HPV	p16/ki-67	Biopsy	Histology	Interval
1	57	ASCUS ASCUS HSIL No cytology	9.0 (HC2)* Other 12+ Other 12+ Other 12+, 608.0 (HC2)	+ + +	1 2 3 4	Negative Negative LSIL CIN2-3	14 months 6 months 20 days
2	35	ASC-H No cytology	1238.0 (HC2)	+	1 2	Negative CIN3	9 months
3	35	HSIL HSIL	24.7 (HC2) 492.6 (HC2)	+	1 2	CIN1 CIN2	9 months
4	27	ASCUS LSIL	501.8 (HC2)	+	1 2	CIN1 CIN2	6 months
5	45	HSIL No cytology	20.4 (HC2)	+	1 2	CIN1 CIN2	25 months
6	56	HSIL No cytology	0.8 (HC2)	+	1 2	Negative CIN3	52 months
7	44	ASC-H No cytology	HPV16+	+	1 2	Negative CIN2	5 months
8	56	LSIL No cytology	Other 12+	+	1 2	Negative VaIN III	22 months
9	55	LSIL No cytology	Other 12+ 1562.0 (HC2)	+	1 2	CIN1 VaINII	19 months

ASC-H, atypical squamous cells cannot exclude high-grade squamous intraepithelial lesion; ASCUS, atypical squamous cells of undetermined significance; CIN, cervical intraepithelial neoplasia HSIL, high-grade squamous intraepithelial lesion; LSIL, low-grade squamous intraepithelial lesion; NILM, negative for intraepithelial lesions or malignancy; VaIN, vaginal intraepithelial neoplasia.

Other 12+: positive for 12 HPV genotypes (HPV 31, 33, 35, 39, 45, 51, 52, 56, 58, 59, 66 and 68), and negative for HPV16/18.

*A relative light unit of 1 (1.0 pg/mL) in HC2 detection was used as the cut off for HR HPV positivity.

## Discussion

Accumulating evidence demonstrated that p16/ki-67 dual-staining cytology showed a high sensitivity and specificity in identifying high grade cervical lesions (10). In the current study, we confirmed that the positive p16/ki-67 dual-staining was associated with HR HPV infection. p16/ki-67 dual-staining is valuable in identifying CIN2+ lesions, regardless of HPV genotype and tissue type, and helps to reduce the missed diagnosis of CIN2+ in HPV negative patients. In particular, the triple detection, p16/ki-67 dual-staining combined with cytology and HPV testing, showed the great advantages in screening CIN2+ lesions. Positive triple detection (positive for all three methods) can effectively predict the possibility of high grade cervical lesions. Our work puts forward and emphasizes the feasibility and importance of triple detection in the screening of cervical lesions or cervical cancer

Compared with HR HPV negative samples, the positive rate of p16/ki-67 dual-staining in HR HPV positive samples is higher, which is reasonable. However, we noted that the positive rate of p16/ki-67 dual-staining was relatively high in HR HPV negative CIN2+ cases (16/23, **Table 2**). The reasons seem complicated. First, this indicated that p16/ki-67 positivity can well reflect the severity of cervical lesions regardless of HPV testing results. Another explanation may be related to techniques. In a previous study, 131 cases underwent HPV genotyping, and 16 cases were found to be infected by HPV types other than the HR HPV, such as HPV53 and HPV73 (33). Among them, four cases were positive for p16/ki-67, and one of the four cases was diagnosed as CIN2. This implied that missed detection of HPV (HPV negative) may be due to specific HPV genotypes not covered by the detection method or low HPV DNA abundance beyond the technical scope (34). This is why the difference of p16/ki-67 dual-staining positive rates between HR HPV positive CIN2+ and HR HPV negative CIN2+ is not always significant when the sample size is not large enough (**Table 2**). These data indicate that p16/ki-67 dual-staining is helpful to reduce the missed diagnosis of CIN2+ in HPV negative patients, highlighting the importance of p16/ki-67 dual-staining in screening cervical CIN2+ lesions, regardless of HPV testing results.

In clinical practice, cervical cytological specimens are usually screened first by LBC or HR HPV testing. Due to the low sensitivity of cytological method and low specificity of HPV primary screening, consequently, p16/ki-67 dual-staining was widely adopted in the stratification of HPV+, HPV+/NILM, ASCUS and LSIL (20, 25, 29). It takes time to collect samples and test again. In this study, we found that triple detection showed great advantages in screening of CIN2+ lesions although its sensitivity is relatively low. First, more than 96% of CIN2+ cases were positive in two or three detection methods. Secondly, as we have shown above, some potential CIN2+ patients can also found by following up the cases with triple detection positive and no obvious histological lesions. These data suggested that patients who lack pathological changes in histology should be referred for additional biopsy or strict follow up when their cytology, HR HPV and p16/ki-67 are positive, even if HR HPV is negative. This is because the lack of histological changes may be due to the miss of lesion tissue. These results further indicated that the use of triple detection in screening of cervical cancer may reduce missed diagnosis. Therefore, we propose to prepare two liquid-based slides from a single cervical cytological specimen, one for Pap staining and the other for p16/ki-67 dual-staining. The residual samples can be used for HR HPV testing. In this way, a sample can be tested in three ways, which effectively shortens the examination time. The whole procedure is also simple and feasible. The results of triple detection can be analyzed simultaneously with the clinical data so as to improve the diagnostic accuracy and reduce the missed diagnosis of CIN2+ lesions. Of course, the cost will increase, but it seems acceptable.

Although the triple detection is simple and valuable in screening high grade cervical lesions, it is mainly based on a retrospective study. Since most participants are patients with high grade cervical lesions, the subject bias may have an impact on the true meaning of the results. In addition, the sample size of this study was not too large. Before promoting this strategy, a comprehensive evaluation carried out in the screening population is needed.

## Data Availability Statement

The original contributions presented in the study are included in the article/**Supplementary Material**. Further inquiries can be directed to the corresponding authors.

## Ethics Statement

The studies involving human participants were reviewed and approved by Ethics Committee of the First Affiliated Hospital, Sun Yet-sen University. The patients/participants provided their written informed consent to participate in this study.

## Author Contributions

LY and SC performed study concept and design, and writing, review and revision of the paper; XC, XL, LF, HM, TT, and LW performed experiments and provided analysis and interpretation of data, and statistical analysis; All authors read and approved the final paper.

## Funding

This study was funded by the National Natural Science Foundation of China (No. 31670788 and No. 81172485) and Open Fund of Guangdong Key Laboratory of Pharmaceutical Functional Genes (No. 2020B1212060031 and No.2017B030314021).

## Conflict of Interest

The authors declare that the research was conducted in the absence of any commercial or financial relationships that could be construed as a potential conflict of interest.

## Publisher’s Note

All claims expressed in this article are solely those of the authors and do not necessarily represent those of their affiliated organizations, or those of the publisher, the editors and the reviewers. Any product that may be evaluated in this article, or claim that may be made by its manufacturer, is not guaranteed or endorsed by the publisher.

## Supplementary Material

Supplementary Table 1 (supplementary to ). Comparison (*p*-value) of sensitivity, specificity, positive predictive value and negative predictive value for CIN2+ cases among different methods. ^a^DSH, p16/ki-67, HR HPV; ^b^CH, cytology, HR HPV; ^c^DSHC, p16/ki-67, HR HPV, cytology. ^d^ Four sets of figure represent the *p*-value of sensitivity, specificity, positive predictive value and negative predictive value, respectively.
