# Supplementary material for: Significance of Triple Detection of p16/ki-67 Dual-Staining, Liquid-Based Cytology and HR HPV Testing in Screening of Cervical Cancer: A Retrospective Study
Source: Front Oncol. 2022 Jun 7;12:915418. doi: 10.3389/fonc.2022.915418 (PMC9209639; doi:10.3389/fonc.2022.915418)
Supplement: Supplementary Table 1 — (supplementary to ). Comparison (p-value) of sensitivity, specificity, positive predictive value and negative predictive value for CIN2+ cases among different methods. aDSH, p16/ki-67, HR HPV; bCH, cytology, HR HPV; cDSHC, p16/ki-67, HR HPV, cytology. d Four sets of figure represent the p-value of sensitivity, specificity, positive predictive value and negative predictive value, respectively. [file Table_1.docx]

**Table S1** (supplementary to Table 3)**.** Comparison (*p*-value) of sensitivity, specificity, positive predictive value and negative predictive value for CIN2+ cases among different methods

|  | p16/ki-67 | Cytology | HR HPV | DSH+ | CH+ | DSHC+ |
| --- | --- | --- | --- | --- | --- | --- |
| p16/ki-67 |  |  |  |  |  |  |
| Cytology | < 0.001, < 0.001, < 0.001, 0.003 ^d^ |  |  |  |  |  |
| HR HPV | 0.001, < 0.001, < 0.001, 0.272 | 0.031, < 0.001, 0.014, 0.002 |  |  |  |  |
| ^a^DSH+ | 0.116, 0.342, 0.686, 0.229 | < 0.001, < 0.001, < 0.001, < 0.001 | < 0.001, < 0.001, < 0.001, 0.759 |  |  |  |
| ^b^CH+ | 0.012, < 0.001, < 0.001, 0.615 | 0.003, 0.002, 0.274, 0.018 | 0.410, < 0.001, < 0.001, 0.159 | < 0.001, < 0.001, < 0.001, 0.101 |  |  |
| ^c^DSHC+ | 0.064, 0.037, 0.186, 0.213 | < 0.001, < 0.001, < 0.001, < 0.001 | < 0.001, < 0.001, < 0.001, 0.780 | 0.780, 0.254, 0.366, 0.967 | < 0.001, < 0.001, < 0.001, 0.092 |  |

^a^DSH, p16/ki-67, HR HPV; ^b^CH, cytology, HR HPV; ^c^DSHC, p16/ki-67, HR HPV, cytology.

^b^Four sets of figure represent the *p*-value of sensitivity, specificity, positive predictive value and negative predictive value, respectively.
